# Bioenergetics of the spinal cord in experimental autoimmune encephalitis of rats

**DOI:** 10.1186/s12868-015-0175-1

**Published:** 2015-06-20

**Authors:** Mariam Al-Shamsi, Allen Shahin, Marwa F Ibrahim, Saeed Tareq, Abdul-Kader Souid, Eric P K Mensah-Brown

**Affiliations:** Department of Medical Microbiology and Immunology, College of Medicine and Health Sciences, United Arab Emirates University, Al Ain, Abu Dhabi UAE; Department of Anatomy, College of Medicine and Health Sciences, United Arab Emirates University, Al Ain, Abu Dhabi UAE; Department of Pediatrics, College of Medicine and Health Sciences, United Arab Emirates University, Al Ain, Abu Dhabi UAE

## Abstract

**Background:**

Mitochondrial dysregulation is important in axonal damage and demyelination in multiple sclerosis (MS) and experimental autoimmune encephalomyelitis (EAE). There is however, no evidence in the literature of any study that has examined cellular bioenergetics of the central nervous system (CNS) during the early development and clinical course of EAE. EAE, a rodent model of relapsing/remitting MS, is a CD4^+^ T cell-mediated disease of the CNS. We hypothesize that CNS bioenergetics might predict prognosis, and that preserved bioenergetics might underlie the remission from disease. The study aims therefore, to determine whether the clinical history of EAE is influenced by cellular respiration of the CNS in susceptible Dark Agouti (DA) and resistant Albino Oxford (AO) rats.

**Methods:**

Experimental autoimmune encephalomyelitis was induced by myelin basic protein in complete Freud Adjuvant in the footpads of DA and AO rats. A phosphorescence analyzer that determines cellular respiration was used to monitor oxygen consumption and ATP concentration was measured using the Enliten ATP assay system. Disease pathology was demonstrated by H&E and Luxol fast blue staining of sections of the lumbar regions of the spinal cord. Mitochondrial size in relation to axonal size was determined by electron microscopy. Apoptosis was studied by HPLC measurement of intracellular caspase-3 activity and caspase immunohistochemistry. Role and source of caspase 1 was studied by double immunofluorescence with antibodies for caspase-1, microglia (anti-Iba1) and astrocytes (anti-GFAP).

**Results:**

The cellular respiration of the CNS did not vary between diseased and normal rats. We also demonstrate here, that at the peak of disease, inflammation as shown by caspase-1, produced by activated microglia and infiltrating cells, was significant in susceptible DA rats. The mitochondrial:axonal size ratio did not vary in the different groups although mitochondria were smaller in spinal cords of diseased DA rats. Demyelination, observed only in areas of mononuclear infiltration of the spinal cord of diseased DA rats, was demonstrated by light microscopy and electron microscopy.

**Conclusion:**

We conclude that EAE at this early stage does not significantly affect CNS cellular respiration and this might underlie the reason for the recovery of diseased rats.

## Background

Cellular bioenergetics which is the biochemical processes involved in energy metabolism (energy conversion or transformation) and cellular respiration (mitochondrial O_2_ consumption), describes the delivery of metabolites and O_2_ to mitochondria, the oxidation of reduced metabolic fuels with passage of electrons to O_2_, and the synthesis of ATP. Impaired cellular bioenergetics or respiration, thus, entails an interference with any of these processes. Work by Mahad et al. [1] and several others have demonstrated the importance of mitochondrial dysregulation and mitochondria-derived reactive oxygen species (ROS) to axonal damage and demyelination in multiple sclerosis (MS) and experimental autoimmune encephalomyelitis (EAE) [1–8]. While the EAE model has been successfully employed to investigate the mechanism of MS, there is no evidence in the literature of any study that has examined cellular bioenergetics of the central nervous system (CNS) during the early development and clinical course of EAE. This study would attempt to provide an explanation for some of the features of EAE, most significant of which is that, diseased rodents apparently recover with minimal neurological deficits. Recovery has been attributed to the downregulation of proinflammatory cytokines resulting from the clearance of infiltrating T cells by activated microglia [9].

Experimental autoimmune encephalomyelitis is a rodent model for relapsing/remitting type of MS [10, 11]. Injection of encephalitogenic emulsion into the footpads of rats induces disease in the genetically susceptible Dark Agouti (DA), but not the resistant Albino Oxford (AO) strains although mild inflammation occurs in both strains early after disease induction [12–14]. The active phase of the MS lesion and EAE is also characterized by the infiltration of T lymphocytes and monocyte-derived macrophages, which initiate demyelination [15, 16]. These cells together with activated microglia have been shown to by several other workers [1, 2, 17, 18] to secreted proinflammatory mediators and oxidizing radicals, such as superoxide anion, hydroxyl radicals, hydrogen peroxide, and nitric oxide (NO), which are believed to be responsible for the demyelination and axonal damage in MS and EAE.

Apoptosis describes highly regulated mechanisms responsible for cellular responses to injuries or biologic signals. Caspases-1 and -3, cysteine aspartate-directed proteases and members of the interleukin-1β-converting enzyme (ICE) group, are the key executors of apoptosis and inflammation [19, 20]. Activation of caspases leads to the opening of the transitional permeability pores in the inner mitochondrial membranes, which results in uncoupling oxidative phosphorylation as well as rapid cellular ATP and nutrient depletion. This process produces deleterious morphological and biochemical changes, including mitochondrial disturbances which may cause cell death. MS and EAE result from apoptosis of oligodendrocytes arising from the effect of inflammation induced by infiltrating mononuclear and resident CNS cells [21–24]. We have in this study investigated spinal tissue respiration (cellular mitochondrial O_2_ consumption) in MBP-induced EAE in rats using a sensitive in vitro system that employs a phosphorescence analyzer to measure O_2_ concentration as a function of time in tissues. We have also measured the sizes of mitochondria and the axons within which they occur, to determine the presence of any morphological evidence of impairment of mitochondrial function. We show here that spinal tissue respiration appears preserved and the differences in the ratio of mitochondrial:axonal sizes are insignificant between the groups studied. Further, we have shown increased levels of apoptosis in diseased DA rats by caspase-3 immunohistochemistry and by measuring the ability of caspase-3 to cleave Ac-DEVD-AMC to release the fluorogenic moiety, 7-amino-4-methylcoumarin (AMC). By means of double immunofluorescence using rabbit anti-caspase 1 either with goat anti- Ionized calcium binding adaptor molecule 1 (Iba-1) for microglia [25] and with mouse anti glial fibrillary acidic protein (GFAP) [26] for astrocytes, we have shown that caspase 1 induced inflammation [27] is important in disease development in EAE and microglia together with other infiltrating mononuclear cells are the source of caspase-1. Activated microglial cells have been shown to mediate axoglial disruption that contributes to axonal damage in multiple sclerosis [28].

Our hypothesis is that CNS bioenergetics might predict prognosis, and that preserved bioenergetics, as observed in this initial wave of EAE, might underlie the remission from disease. Any intervention, therefore, that sustains cellular energy metabolism during the course of EAE would be expected to improve disease outcome.

## Methods

### Animals

Male Dark Agouti (DA) and Albino Oxford (AO) rats (8–12-week-old) weighing between 250 and 300 g used in this study were maintained at the animal facility of the College of Medicine and Health Sciences, UAE University in compliance with NIH guidelines (http://grants.nih.gov/grants/olaw/references/phspol.htm). Although female rats are generally more susceptible to autoimmunity, male rats were used in this study because previous studies by Lenz et al. [29] and others [13, 14, 30] since then, have revealed no gender differences in disease susceptibility. All rats were housed in rooms maintained at 22°C with ~60% relative humidity and a 12-h light/dark cycle. All rats had ad libitum access to standard rodent chow and filtered water. All protocols used here received approval from the Animal Research Ethics Committee, United Arab Emirates University.

For these studies, experimental rats were anaesthetized by intra-peritoneal injection of 1.5 ml/100 g (BW) of urethane [using 20% solution (w/v) in 0.9% NaCl].

### Reagents

The Pd(II) complex of *meso*-tetra-(4-sulfonatophenyl)-tetrabenzoporphyrin (Pd phosphor) was obtained from Porphyrin Products (Logan, UT, USA). Dactinomycin (actinomycin D, MW ≈ 1,255) was purchased from Merck (Whitehouse Station, NJ, USA). A lyophilized powder of caspase inhibitor I (zVAD-fmk, MW ≈ 467.5) was purchased from Calbiochem (La Jolla, CA, USA). Ac-DEVD-AMC (MW ≈ 729.6) and caspase-3 (molecular mass ≈ 30.5 kDa, heterodimer active human recombinant) were purchased from Axxora LLC (San Diego, CA, USA). Glucose [anhydrous], bovine serum albumin (free of endotoxin and fatty acids), and remaining reagents were bought from Sigma-Aldrich (St. Louis, MO, USA).

Dactinomycin solution was made fresh in dH_2_O; its concentration was determined by absorbance at 440 nm, using an extinction coefficient of 24,450 M^−1^ cm^−1^ [31]. The zVAD-fmk solution (2.14 mM) was made by dissolving 1.0 mg of zVAD-fmk in 1.0 ml of dimethyl sulfoxide and stored at −20°C. The Ac-DEVD-AMC caspase substrate was dissolved in dimethyl sulfoxide at a concentration of 6.85 mM and stored at −20°C in small aliquots. Phosphate-buffered saline (PBS) with glucose (137 mM NaCl, 2.7 mM KCl, 4.3 mM Na_2_HPO_4_, 1.4 mM KH_2_PO_4_, and 5 mM glucose; pH 7.4) was made fresh. NaCN solution (1.0 M) was prepared in dH_2_O; the pH was adjusted to ~7.0 with 12 N HCl and stored at −20°C.

### Induction of EAE

Groups of DA and AO rats (n = 5) were immunized in the left hind foot pad with 0.1 ml antigenic emulsion containing rat spinal cord tissue and 100 μg of rat myelin basic protein (MBP) with complete Freund’s adjuvant (CFA) (both obtained from Sigma-Aldrich, St Louis, MO, USA). Animals were monitored for clinical disease starting from day 4 after immunization. The severity of disease was assessed by grading tail, hind limb and forelimb weakness, each on a scale of 0–4 [12, 13]. Briefly, zero indicated no disease, 1—loss of tail tonicity, 2—hind limb weakness, 3—hind limb paralysis, and 4—moribund or death. Spinal cord specimens of the lumbar region of immunized DA and AO rats were excised at induction, peak of disease and resolution phases of the disease. Spinal cord specimens were also collected from non-immunized DA rats. For analysis, DA rats at peak of disease (grade 3+) which occurred on days after immunization which varies (9–14 days) were used.

### Excision of spinal cord tissue

Spinal cord specimen (18–30 mg/rat) were excised from the lumbar region using a sharp scalpel blade. Following protocols previously outlined [32, 33], the specimens were *immediately* immersed in ice-cold oxygenated Krebs–Henseleit buffer [115 mM NaCl, 25 mM NaHCO_3_, 1.23 mM NaH_2_PO_4_, 1.2 mM Na_2_SO_4_, 5.9 mM KCl, 1.25 mM CaCl_2_, 1.18 mM MgCl_2_, and 6 mM glucose (pH 7.2)], weighed and then placed in 1.0 ml Krebs buffer containing 0.5% fat-free bovine albumin and 3 μM Pd phosphor for O_2_ measurements. Unless otherwise noted, the time period between specimen collection and start of O_2_ measurement was <5 min. Where stated, specimens were incubated in vitro at 37°C in Krebs–Henseleit solution gassed with 95% O_2_:5% CO_2_ prior to O_2_ measurements.

### Oxygen measurements

A phosphorescence oxygen analyzer was used to monitor O_2_ consumption by spinal cord specimens [32, 33]. O_2_ detection was performed with the aid of Pd phosphor that had an absorption maximum at 625 nm and a phosphorescence maximum at 800 nm. Samples were exposed to light flashes (600 per min) from a pulsed light-emitting diode array with peak output at 625 nm (OTL630A-5-10-66-E, Opto Technology, Inc., Wheeling, IL, USA). Emitted phosphorescent light was detected by a Hamamatsu photomultiplier tube (928) after first passing it through a wide-band interference filter centered at 800 nm. The amplified phosphorescence decay was digitized at 1.0 MHz by a 20-MHz A/D converter (Computer Boards, Inc., Mansfield, MA, USA).

A program was developed using Microsoft Visual Basic 6, Microsoft Access Database 2007, and Universal Library components (Universal Library for Measurements Computing Devices; http://www.mccdaq.com/daq-software/universal-library.aspx). It allowed direct reading from the PCI-DAS 4020/12 I/O Board (PCI-DAS 4020/12 I/O Board; http://www.mccdaq.com/pci-data-acquisition/PCI-DAS4020-12.aspx). The pulse detection was accomplished by searching for 10 phosphorescence intensities >1.0 V (by default). Peak detection was accomplished by searching for the highest 10 data points of a pulse and choosing the data point closest to the pulse decay curve [34].

The phosphorescence decay rate (1/τ) was characterized by a single exponential; I = Ae^−*t*/τ^, where I = Pd phosphor phosphorescence intensity. The values of 1/τ were linear with dissolved ${\text{O}}_{ 2}:{ 1}/\tau = 1/\tau^\circ + k_{q} \left[ {{\text{O}}_{ 2} } \right]$, where 1/τ = the phosphorescence decay rate in the presence of O_2_, 1/τ° = the phosphorescence decay rate in the absence of O_2_, and *k*_q_ = the second-order O_2_ quenching rate constant in s^−1^ μM^−1^ [35].

Spinal cord tissue respiration was measured at 37°C in 1 ml sealed vials. Mixing was carried out with the aid of parylene-coated stirring bars. In vials sealed from air, [O_2_] decreased linearly with time, indicating the kinetics of mitochondrial O_2_ consumption was zero-order. The rate of respiration (*k*, in μM O_2_ min^−1^) was thus negative of the slope d[O_2_]/d*t*. Sodium cyanide (NaCN) inhibited respiration, confirming that O_2_ was being consumed in the mitochondrial respiratory chain.

Calibration with β-glucose plus glucose oxidase: The calibration reaction contained PBS with 3 μM Pd phosphor, 0.5% fat-free albumin, 50 mg ml^−1^ glucose oxidase and various concentrations of β-glucose. The values of 1/t were linear with [β-glucose]; the value of *k*_q_ was the negative of the slope (*k*_q_ = 101.1 s^−1^ μM^−1^). The value of 1/τ for air-saturated solution (without glucose) was 28,330 s^−1^ (coefficient of variation, C_v_ = 12%) and for O_2_-depleted solution (with 500 mM β-glucose, 1/τ_o_) 2,875 s^−1^ (C_v_ = 1%). The high values of C_v_ for the air-saturated solutions were due to the lower phosphorescence intensities with high [O_2_] (little light reaching the photomultiplier tube). O_2_ concentration was calculated using, 1/τ = 1/τ° + *k*_*q*_[O_2_] [35].

Dissolved O_2_ is expressed in mm Hg, ml O_2_ l^−1^, mg O_2_ l^−1^, or mmol l^−1^ (mM). For conversion: a partial pressure of oxygen (*P*O_2_) of 1.0 mm Hg = 0.03 ml O_2_ l^−1^; 1.0 ml O_2_ l^−1^ = 1.4276 mg O_2_ l^−1^; 1.0 mg O_2_ l^−1^ = 1,000/32 μM. In freshwater at 760 mm Hg and 20°C, dissolved [O_2_] is 9.1 mg l^−1^, or 284 μM. Using a Clark electrode, the *P*O_2_ of our reaction mixture (PBS with 10 mM glucose, 3.0 μM Pd phosphor and 0.5% fat-free bovine serum albumin) was 170.5 [±6.6] mm Hg (n = 4), or 228 [±9] μM. The 56 mm Hg difference between [O_2_] in freshwater and the Pd solution reflects the effect of salinity on dissolved O_2_.

### Intracellular caspase-3 activity measurement by HPLC

Ac-DEVD-AMC is a synthetic substrate that enters cells rapidly and is cleaved by caspases to yield the fluorescent compound, 7-amino-4-methylcoumarin (AMC). Following cell disruption, any released AMC is separated by HPLC and detected by fluorescence. The use of the pan-caspase inhibitor zVAD-fmk results in a reduction in AMC peak area confirming that intracellular caspases are responsible for the cleavage reaction. Specimens (~40 mg) were incubated in oxygenated KH buffer containing 74 μM Ac-DEVD-AMC with or without 43 μM zVAD-fmk (Calbiochem, La Jolla, CA, USA) for 30 min (final volume, 0.5 ml) at 37°C and processed as previously reported [16]. Briefly, the tissue was homogenized by 10 passages through a 27-G needle and the Ac-DEVD-AMC (*N*-acetyl-asp-glu-val-asp-7-amino-4-methylcoumarin; *m.w.* = 675.64; caspase-3 substrate, Axxora LLC San Diego, CA, USA) cleavage reaction was quenched with dilution, a procedure which renders caspases inactive. The supernatant was collected by centrifugation (16,300*g* for 90 min) and separated on HPLC Waters 1525 reversed-phase HPLC system (Spectra Lab Scientific Inc, Alexandria, VA, USA), and analyzed for the free fluorogenic AMC moiety [30]. The excitation wave length used was 380 nm with an emission wave length of 460 nm. zVAD-fmk solution (2.14 mM) was made by dissolving 1.0 mg in 1.0 ml dimethyl sulfoxide and stored at −20°C. Ac-DEVD-AMC solution (7.4 mM) was made by dissolving 5.0 mg in 1.0 ml dimethyl sulfoxide and stored at −20°C.

### ATP content measurement

Spinal cord fragments were homogenized in ice-cold 2% trichloroacetic acid for 2 min and neutralized with 100 mM Tris–acetate, 2 mM EDTA, pH 7.75. The supernatant was collected by centrifugation (1,000×*g* at 4°C for 5 min) and stored at −20°C until analysis. The pH of samples was adjusted to 7.75 immediately before ATP determination. ATP concentration was measured using the Enliten ATP Assay System (Bioluminescence Detection Kit, Promega, Madison, WI, USA). Briefly, 2.5 μl of the acid-soluble supernatant was added to 25 μl of the luciferin/luciferase reagent. The luminescence intensity was measured at 25°C using Glomax Luminometer (Promega, Madison, WI, USA). The ATP standard curve was linear from 10 pM to 100 nM (*R*^2^ > 0.9999).

### Histological analysis

Spinal cord specimens (n = 5) from immunized and non-immunized animals were fixed in 4% phosphate-buffered paraformaldehyde by perfusion and routinely embedded in paraffin wax. Sections of thickness 5–7 µm were then stained with hematoxylin & eosin for light microscopy. The level of mononuclear cell infiltration was graded using the following semi-quantitative scoring: 0, no infiltration; 1, mild infiltration around pial vessels; 2, single-cell infiltration within the CNS; 3, infiltration with mild perivascular cuffing; and 4, very intense infiltration with perivascular cuffing [12, 13].

### Luxol fast blue microscopy

Procedures recommended by the manufacturers were applied. Briefly, 5–7 μm thick sections of paraffin wax embedded spinal cords from immunized DA (grade 3 disease) and AO rats were deparaffinized in two changes of xylene for 10 min, hydrated in changes of series of 100 and 95% alcohol. The sections were incubated in Luxol Fast Blue (LFB) solution in 56°C oven overnight. After rinsing off excess LFB with 95% alcohol and distilled water, sections were differentiated in lithium carbonate solution and 70% alcohol for 30 s each, followed by a final rinse in distilled water. After checking for completion of differentiation under the microscope, sections were counterstained in cresyl violet solution for 1–2 min, covers lipped with DPX and examined under the Zeiss axiophot microscope to which a digital camera is attached.

### Immunohistochemistry

To determine the presence and source of caspase-1, 5–7 μm thick sections of paraffin wax embedded spinal cords from immunized DA (grade 3+ disease) and AO rats were stained by double immunofluorescence polyclonal rabbit anti-caspase-1 (Abcam, Cambridge, USA) and Iba-1 and caspase-1 and GFAP and Goat polyclonal anti-ionized calcium-binding protein (Iba-1; Wako, Germany) which unlike other microglial markers is expressed by all subpopulations of microglia [25] and anti-mouse glial fibrillary acidic protein (GFAP; Vector Laboratories Inc., CA, USA) for astrocytes [26]. Briefly, rehydrated sections were transferred into 0.1 M citrate buffer and boiled in a 750 W microwave for antigen retrieval. The sections were incubated with the antibodies diluted 1:200 in BSA/0.1 M PBS overnight at 4°C. Control sections were treated with antibodies preadsorbed with peptides provided from the same sources as antibodies at a concentration of 10^−6^ M. On the following morning, sections were washed 3× for 5 min, incubated with the link antibody comprising fluorescein isothiocyanate (FITC) bound anti-rabbit IgG and rhodamine (RRX) bound anti-goat or mouse IgG (Jackson ImmunoResearch Laboratories Inc., USA) diluted 1:100 in BSA/0.1 M PBS for 1 h at room temperature. Sections were coverslipped with immunomount (Shandon, Pittsburgh, USA) and examined by a Nikon Eclipse 80i confocal microscopy with Nikon D Eclipse C1 single photon laser probe.

### Quantification of caspase-3 immunoreactive cells

Non-contiguous sections of the spinal cord taken from the lumbar region (L3) were used for quantification. The total number of caspase-3 immunoreactive cells per section present in three non-contiguous sections (every 5th section) of the spinal cord of immunized DA (grade 3 diseased; n = 5) and AO rats was obtained by counting using a ×20 objective on a Zeiss axiophot photomicroscope.

### Electron microscopy

Sections of the spinal cord from immunized DA (n = 4, grade 3–4 disease), non-immunized DA (n = 4) and immunized but resistant AO rats (n = 4) were fixed by perfusion with freshly prepared Karnovsky’s solution. Sections from the lumbar region were then further fixed by immersion in Karnovsky’s solution for 18 h and routinely processed for electron microscopy. Thick sections (1–2 μm) from corresponding areas of the spinal cords of the different groups of rats were stained with toluidine blue and examined under the light microscope to confirm the areas for electron microscopy. Ultrathin sections of 60–90 nm thickness were then cut with a Delaware diamond knife (Agar Scientific Ltd., Cambridge, UK) and mounted on 3 mm 200 carbon grids. The grids were contrast-stained with saturated aqueous uranyl acetate and lead citrate and examined with the Phillips CM10 transmission electron microscope. Thin sections were taken from areas of the spinal cord showing mononuclear cellular infiltration in diseased DA rats. For comparison similar areas of the spinal cords of immunized AO and non-immunized DA rats were also examined by electron microscopy.

### Quantification of mitochondrial and axonal sizes

A total of three non-contiguous grids from four immunized diseased DA, non-immunized DA and AO rat spinal cords (2 per rat) were analyzed. Sections were examined at ×27,000 magnification and the perimeter of each mitochondrion and that of the axon within which it occurred measured using the Image J analysis software. The ratio of mitochondrial size to that of the axon was then determined for each group of immunized DA and AO rats. Measurements were taken only if the whole profile of the axon could be seen.

### Statistical analysis

Data were analyzed using SPSS statistical package (version 19). The nonparametric test (2 independent variables) Mann–Whitney was used to compare readings of immunized and non-immunized samples.

## Results

**Figure 1:**
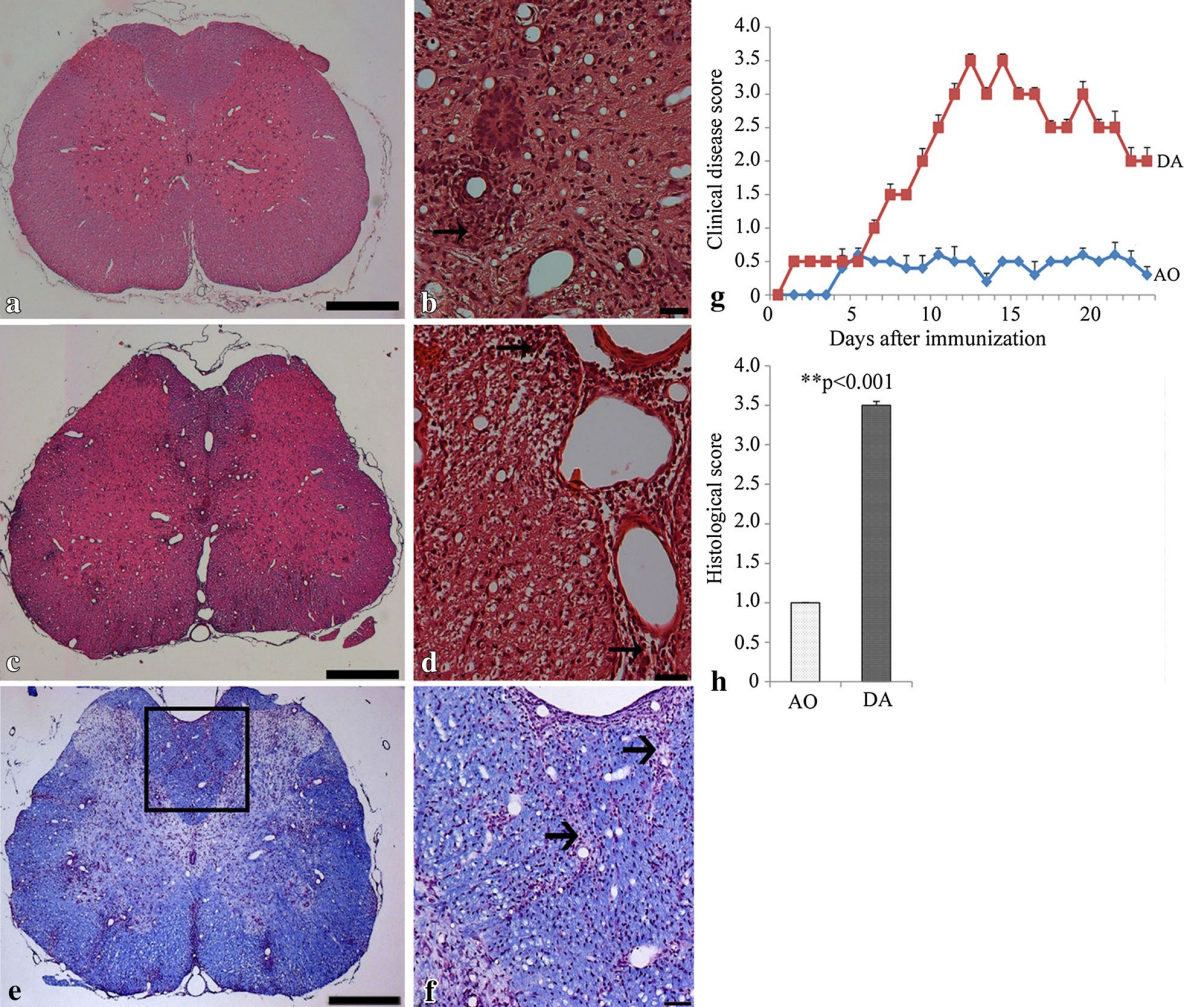
DA rats develop EAE while AO rats are resistant. DA (**b**–**d**) developed severe EAE whilst AO rats did not (**a**). Rats were immunized with MBP in complete Freund adjuvant. **a**, **c** Low magnification micrographs of sections of the spinal cord of AO and DA rats respectively; **b**, **d** high magnification micrographs of spinal cord sections of DA rats examined 14 days after immunization using H&E staining. Note the presence of typical perivascular and subpial infiltrates of different sizes in areas of the spinal cords of DA rats (**b**, **d**). **g** Clinical scores show that DA rats become severely diseased with time, whereas AO rats do not develop the disease. Results are mean clinical scores ± SEM (n = 5, representative of four experiments, p < 0.001). **h** A cumulative graph of semiquantitative assessment of the histological score of disease from the spinal cords of DA and AO rats of 10 noncontiguous sections per spinal cord (p < 0.001, n = 3). **e** Micrographs of Luxol fast blue-stained sections of the spinal cord of immunized DA rats at peak of disease (n = 5). The dorsal column (*square box*) of the DA rat is enlarged in **f** to show areas of infiltration around which there seem to be evidence of demyelination (*arrows*). *Bar* 20 μm.

As previously reported [13, 14], AO and DA rats react differently to disease induction as evaluated by clinical criteria in that while DA rats are susceptible, AO rats are resistant to disease induction. DA and AO rats were immunized in the foot pads with MBP in complete Freund’s adjuvant. Disease development in DA rats normally became evident by day 10 and reached the peak around day 14. AO rats never developed the disease (Figure 1a). The difference in disease scores was confirmed by histopathological analysis of the spinal cord, with the DA rats showing significantly severe inflammation and the presence of perivascular cuffing at the peak of disease (Figure 1b–d). There was a significant difference in the incidence of the disease (Figure 1g) and this was confirmed by quantitative measurement of the histological scores (Figure 1h). As shown in Figure 1e, f, evidence of demyelination as demonstrated LFB staining [36, 37] was minimal and detectable only around areas of mononuclear cellular infiltration of the cord in DA rats at the peak of clinical disease.

### Cellular respiration and ATP content

**Figure 2:**
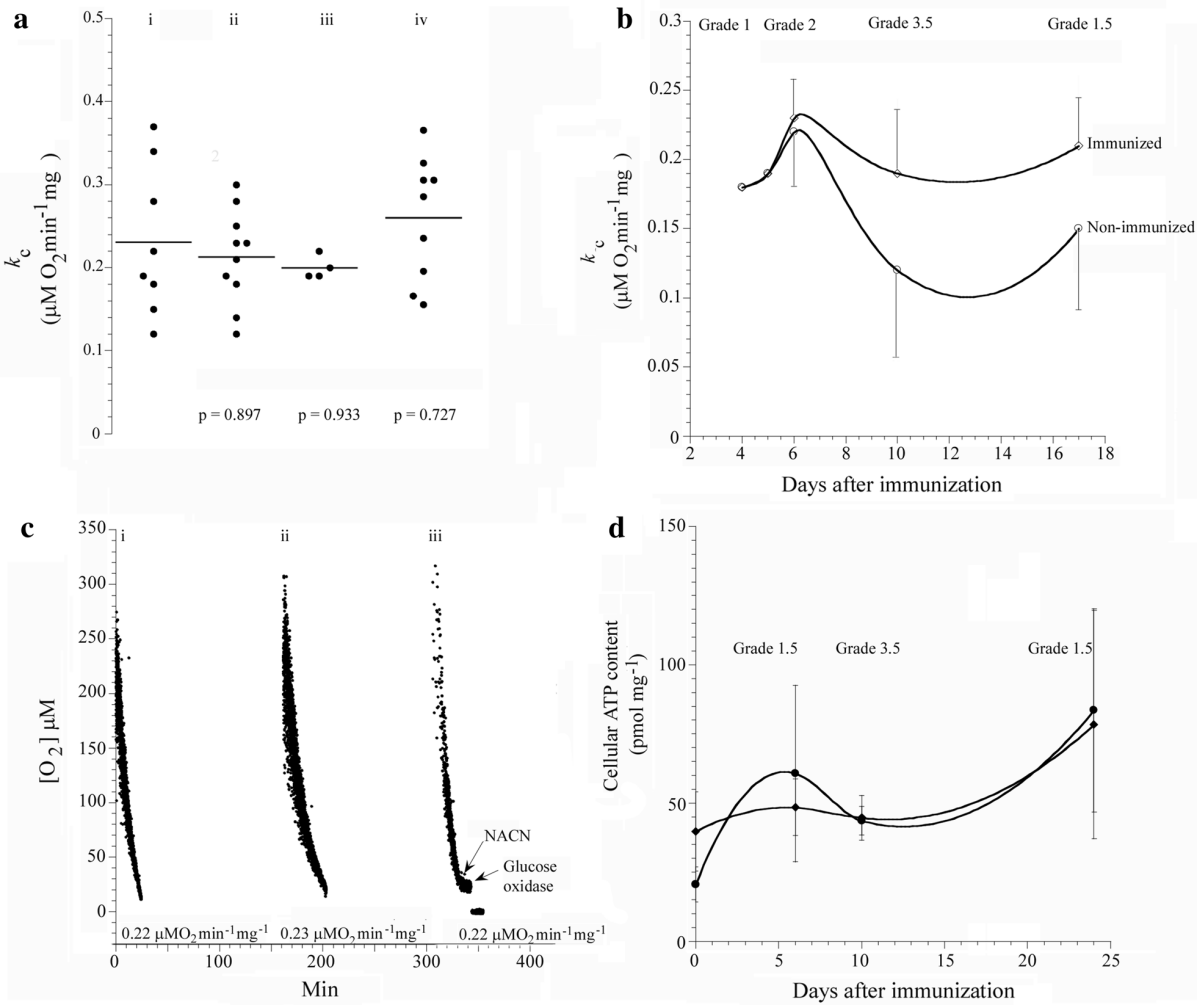
Spinal cord tissue respiration in immunized and non-immunized rats. **a** A summary of all *k*_*c*_ values (involving 33 rats) as a function disease grade for (*i*), immunized DA with grade 1–2 disease (*ii*), immunized DA rats with grade 3+ disease (*iii*) and immunized but resistant AO rats (*iv*). **b** Representative experiment of cellular respiration in immunized and non-immunized DA rats with different grades of disease showing the values of *k*_*c*_ as a function of days after immunization. **c** Representative runs of O_2_ consumption by spinal cord tissue from non-immunized (*i*), immunized with grade 1-2 disease (*ii*) and immunized with grade 3+ disease (*iii*) DA rats. Addition of 10 mM NaCN and 50 μg ml^−1^ glucose oxidase are shown. Rates of respiration (*k*_*c*_, in μM O_2_ min^−1^ mg^−1^) are shown at the *bottom* of the runs. **d** Cellular ATP content in immunized DA and AO rats on days 6, 10 and 24 after immunization. The *error bars* are standard deviations (n = 3 independent samples).

O_2_ consumption by spinal cord tissue was measured using excised specimens from immunized and non-immunized DA rats at different stages of the disease. In addition, O_2_ consumption was compared between immunized DA and AO rats. Respiration was inhibited by cyanide, confirming that oxidation had occurred in the mitochondrial respiratory chain. We demonstrate here that the differences in the rate of respiration (*k*_c_, in μM O_2_ min^−1^ mg^−1^) between non-immunized and immunized DA rats at the inductive phase of grades 1–2 (*p* = 0.897) and peak phase of grades >3 (*p* = 0.933) were not significant. The differences in the values of *k*_c_ between non-immunized and immunized AO rats, which did not develop disease, were also not significant (*p* = 0.722) (Figure 2a–c). Thus clearly spinal cord respiration was not diminished during the course of disease. Consistently, cellular ATP (expressed in pmol mg^−1^) was also preserved throughout the course of disease in immunized DA rats (Figure 1d) with no significant differences between that of immunized diseased DA and immunized AO rats at the different clinical stages of the disease (Figure 2d). There were no significant differences in cellular ATP content between immunized and non-immunized DA rats (data not shown).

### Caspase-3 and caspase-1 activities are both elevated in diseased DA rats

**Figure 3:**
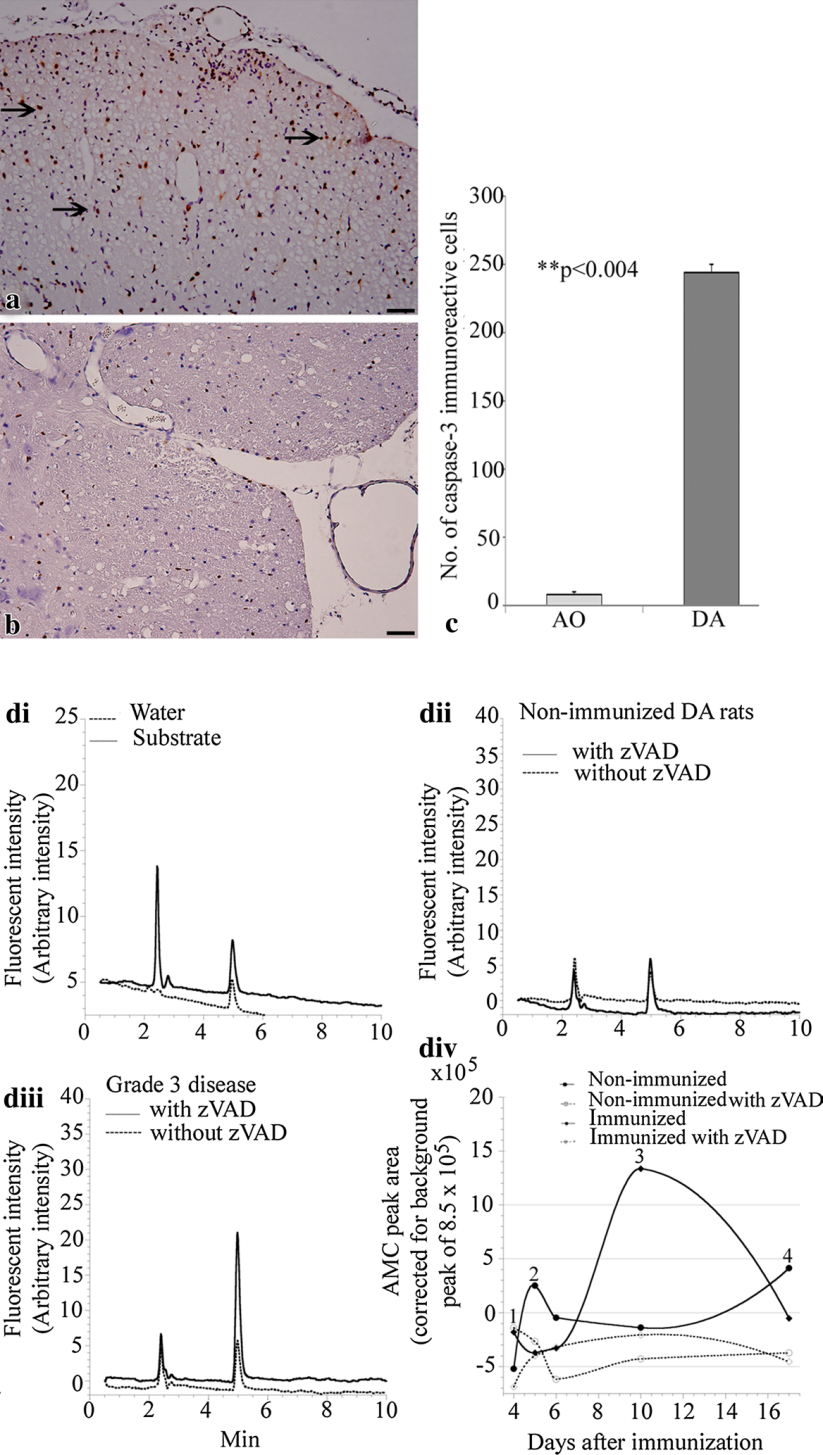
Spinal cord caspase activity in immunized and non-immunized DA rats. A representative micrograph of sections of the spinal cord from diseased DA rats (**a** grade 3.5; n = 4) and immunized but resistant AO (**b**) rats (n = 4) immunostained with polyclonal rabbit anti-caspase-3. Caspase-3 positive cells (*arrows*) were observed only in DA rats. **c** A graph of the number of caspase-3 immunoreactive cells in sections of spinal cords. Spinal cord specimens were also incubated in oxygenated KH buffer plus Ac-DEVD-AMC with and without zVAD-fmk. The tissues were then disrupted by homogenization and the supernatants were separated on HPLC and analyzed for AMC peak. **di** depicts representative runs of dH_2_O and substrate injections, **dii** runs of spinal cord tissue caspase activity of non-immunized DA rat and **diii** representative runs of spinal cord tissue caspase activity of immunized DA rat at the peak of clinical disease. **div** AMC peak areas were plotted as a function of days after immunization in DA rats. A peak, indicative of caspase activity is significant only in DA rats at the peak of clinical disease. This is further confirmed in graphs of AMC peak areas as a function of days after immunization (*complete lines*). Note how caspase activity rises from induction (*1*), grade 2 disease (*2*), till peak of clinical disease (*3*) and recovers (*4*) to levels similar to that of (*1*) after recovery. This is not observed in resistant AO rats (*dotted lines*). *Bar* 25 μm.

**Figure 4:**
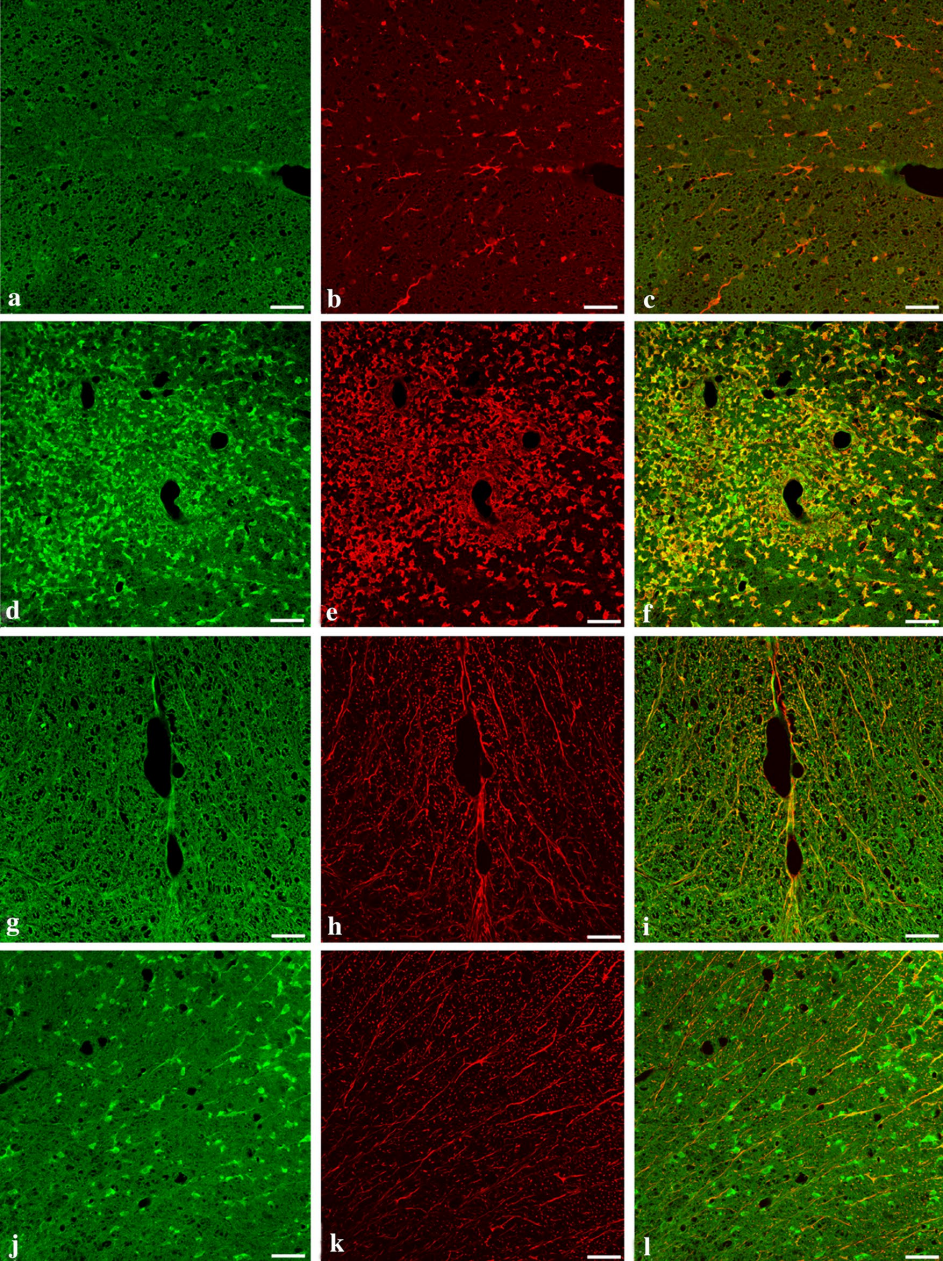
Caspase-1 immunoreactivity is present in infiltrating mononuclear cells and microglia.* Panels* show representative cryostat sections of spinal cord from the lumbar region of AO (**a**–**c**, **g**–**i**) and DA rats at the peak of disease (**d**–**f**, **j**–**l**) 15 days after immunization (n = 4). Micrographs **a**, **d**, **g** and **j**, have been stained by indirect double immunofluorescence with polyclonal rabbit anti caspase-1 (FITC-green), **b**, **e** with goat anti-Iba-1, and **h**, **k** with monoclonal mouse anti-GFAP (both RRX-red) and examined by confocal microscopy. Note the intense immunoreactivity to caspase-1 (**d** infiltrating mononuclear cells) and Iba-1 (**e** activated microglia) and their co-localization in diseased DA rats (**f**). Note also that caspase 1 is absent in GFAP-immunoreactive astrocytes (**l**). *Bar* 10 μm.

Caspases are a family of endoproteases that provide critical links in cell regulatory networks controlling inflammation and cell death [20]. By means of caspase-3 and caspase-1 immunoreactivity, we have studied the roles of apoptosis and inflammation in encephalitogen-induced EAE. We first determined whether there was any difference in caspase-3 activity in spinal cord tissue of immunized diseased DA and immunized resistant AO rats at the peak of disease by immunohistochemistry and demonstrate here in Figure 3a–c, that there were significantly more caspase-3 immunoreactive cells in spinal cords of diseased DA rats compared to that in resistant AO rats (244 ± 6 vs 8 ± 2, p < 0.0004). We then proceeded to monitor caspase-3 activity during the pathology of EAE using immunized DA rats at different stages of disease. This was compared with activity in non-immunized DA rats using an assay which detects by fluorescence, the presence of 7-amino-4-methylcoumarin (AMC). This fluorogenic moiety was separated by HPLC from supernatants of homogenized spinal cord tissue (retention time, *R*_t_, ~5.2 min). Representative runs of caspase activity at the peak of disease (grade 3–3.5) of immunized as well as of non-immunized animals are shown in Figure 4a–c respectively. When the AMC peak areas [corrected for background peaks of dH_2_O (area = 451,431 ± arbitrary units) and substrate injections (area = 844,792 ± arbitrary units)] as a function of days after immunization were plotted, the free AMC moiety was noted only at the peak of disease, with a peak area of 1,336,600 ± arbitrary units (Figure 3di–iv). In all runs, zVAD-fmk blocked AMC peak, confirming the Ac-DEVD-AMC cleavage reaction was mediated by caspases. Thus, the treatment with encephalitogen resulted in increased caspase activity only at the peak of disease. These results are consistent with the above noted preserved spinal tissue respiration and ATP content during the entire course of EAE.

We also investigated whether the differences in the pathology of EAE in the two species was also evident in the level of neuroinflammation as demonstrated by the expression of caspase-1 immunoreactivity in the spinal cords of immunized DA and AO rats. We demonstrate here that caspase 1 immunoreactivity was significantly upregulated in DA rats. We also show here by double immunofluorescence that caspase-1 is co-localized with both mononuclear cells and activated microglia found in diseased DA rats (Figure 4f) but not with ramified resting microglia (Figure 4f) in AO rats nor in astrocytes in both diseased DA and resistant AO rats (Figure 4i, l). As expected, activation of microglia and immunoreactivity with caspase-1 both disappear in DA rats when EAE resolves (figure not shown).

### Electron microscopy

**Figure 5:**
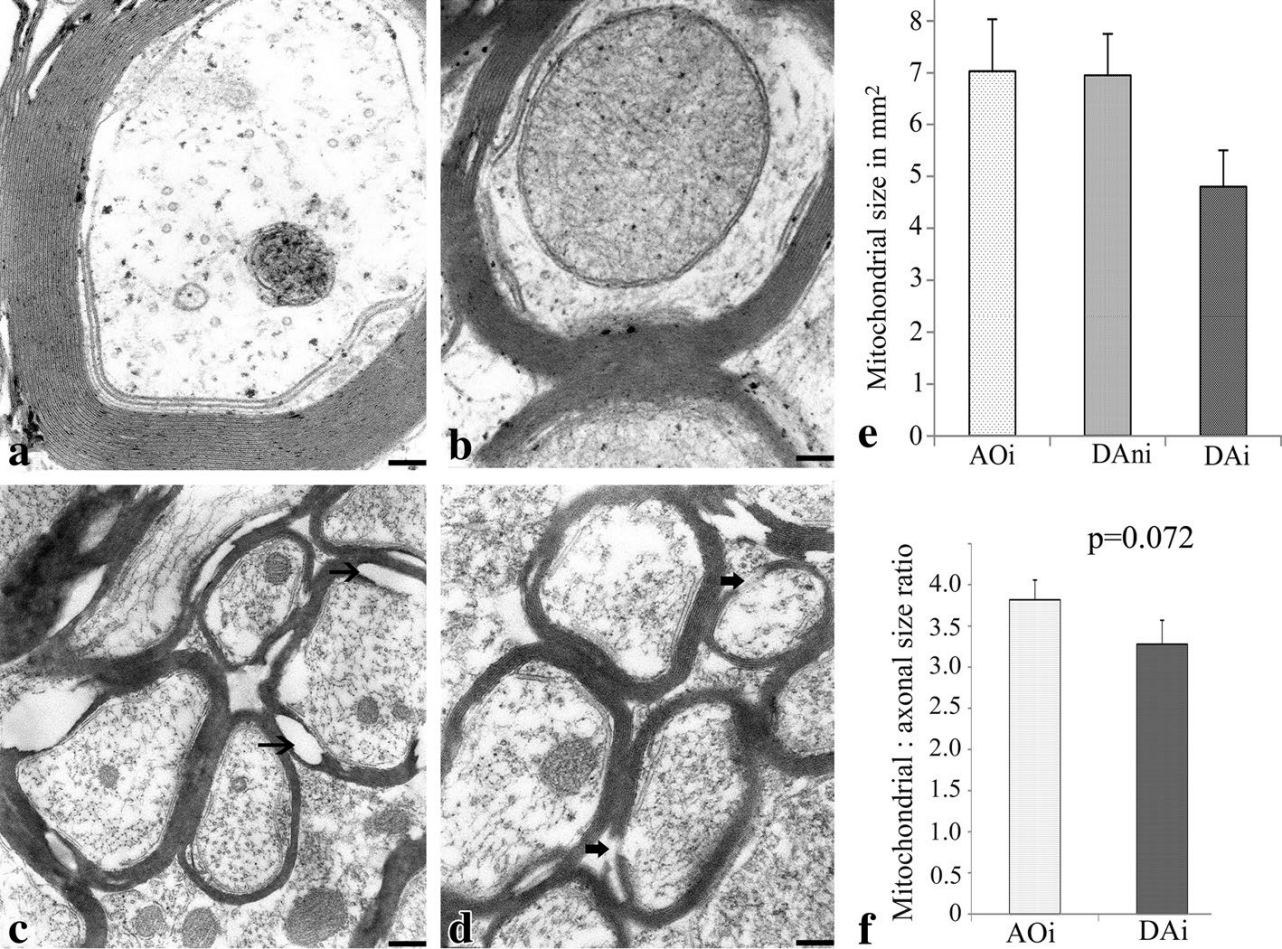
Ultrastructural features of nerve fibres of rats at the peak of clinical disease of EAE. Electron micrographs of sections of the spinal cord of non-immunized DA (**a**), immunized AO (**b**), and grade 3 diseased DA (**c**, **d**) rats (n = 4; 3 non-contiguous grids). Note the normal myelination of fibres in spinal cords of both non-immunized DA and immunized AO rats. Note also the evidence of demyelination in **c** and **d** (*thin arrows*) including splitting of the myelin sheath either at the major dense line or at the intraperiod line. Also present are areas where there seem to be stripping of the nerve fibres of myelin (*thick arrows*). **e** A graph comparing the size of mitochondria in AO with that in DA spinal cords. Note that as shown in **e**, despite the normal appearance of mitochondria in axons of diseased DA rats (*DAi*), they are significantly smaller (p < 0.005) compared with those in the axons of immunized AO (*AO*) and non-immunized DA (*DAni*) rats. Note also that there is no significant difference in the ratio of the mitochondrial:axonal sizes in the immunized diseased DA and resistant AO rats (p = 0.08; **f**). *Bar* **a**, **b** 0.1 μm; **c**, **d** 0.5 μm.

We further examined areas of the white mater of the spinal cords of AO and DA rats by electron microscopy to confirm the demyelination observed by LFB staining and to determine whether this was associated with any damage of mitochondria within both demyelinated and undamaged axons. The effect of EAE on mitochondrial size was assumed to be a measure of the functional status. As shown in (Figure 5a–d), myelin fibers around nerve fibres in the spinal cords of DA rats showed significant evidence of demyelination including splitting of the myelin sheath either at the major dense line or at the intraperiod line and stripping of nerve fibres. Myelin around some nerves were partially lost or seemed to be in the process of being lost. These observations were absent in non-immunized normal DA and immunized but resistant AO rats. Unexpectedly, although they appeared normal in structure, mitochondria from spinal cords of diseased DA rats (4.8 ± 0.7 × 10^9^ mm^2^) were significantly smaller compared with those from non-immunized and normal DA (6.95 ± 0.8 × 10^9^ mm^2^) and immunized but resistant AO rats (7.03 ± 1 × 10^9^ mm^2^; p < 0.001; Figure 5e). It is noteworthy that in spite of the differences in sizes of mitochondria, the difference in the ratio of mitochondrial size:axonal size in diseased DA rats and the immunized AO rats was not significant (3.28 × 10^−6^ ± 0.29 × 10^−6^ vs 3.82 × 10^−6^ ± 0.24 × 10^−6^; p = 0.072; Figure 5f).

## Discussion

Experimental autoimmune encephalomyelitis, a rodent model for relapsing/remitting type of multiple sclerosis in humans, is a T cell mediated disease of the CNS characterized by local inflammatory infiltrates of myelin antigen-specific T cells and demyelination. DA rats are highly susceptible to EAE, whereas AO rats are resistant to disease induction [13, 14] even after immunization with CFA and spinal cord homogenate [4], although mild inflammatory infiltrates are observed in both strains early after disease induction [23]. The cellular and inflammatory infiltrates responsible for the different stages of EAE have been identified to include myelin antigen-specific T cells, B cells, as well as macrophages/monocytes, neutrophils and dendritic cells [24, 36, 38]. The natural history of the disease has therefore been extensively studied, but how these cells influence spinal cord tissue bioenergetics is yet to be elucidated.

In this study, we have applied a highly sensitive analytical method that measures cellular bioenergetics (cellular mitochondrial O_2_ consumption and intracellular caspase activity) to describe the cellular bioenergetics during the course of EAE and to correlate the results with the pathology of the disease. The data show for the first time that spinal tissue respiration and ATP content are preserved throughout the first wave of EAE.

Ac-DEVD-AMC is a synthetic substrate that enters cells rapidly and is cleaved by caspases to yield the fluorescent compound, AMC. Following cell disruption, any released AMC is separated via HPLC and detected by fluorescence. The reduction in AMC peak area due to the pancaspase inhibitor zVAD-fmk confirms that intracellular caspases are responsible for the cleavage reaction. As shown in Figure 3, the AMC peak was first noted at the peak of clinical disease (grade 3.5) and was significantly reduced by zVAD-fmk. These observations correlated with the morphological evidence that anti-caspase-3 immunoreactivity was most significantly increased at the peak of clinical disease. Therefore, the data clearly shows and confirms that the enhanced caspase-3 activity plays a significant role in the development of clinical disease [3].

Multiple sclerosis and experimental autoimmune encephalomyelitis have both been attributed to the loss of oligodendrocytes by apoptosis [39, 40] and hence the probable source of caspase-3 but why and how the disease resolves in rodents remains to be elucidated. Further studies are needed to precisely elucidate the cellular bioenergetic profile of spinal cord tissue at the induction, peak and resolution phases of EAE. Such a task would require separating and studying the different cell populations at these phases of EAE. Nevertheless, the important contribution here is that despite prominent intracellular caspase-3 activity at the peak of disease in DA rats, regardless of its source, the cellular bioenergetics remains preserved. The preserved spinal cord tissue metabolic energy in EAE is consistent with the usual clinical recovery observed in animals. Further, we have demonstrated the heightened inflammation at the peak of disease as evidenced by the increased production of caspase-1 by mononuclear cells and activated microglia [27, 28, 41–45] at the peak of disease. Therefore this increased caspase activity and inflammation seem to occur probably with very little effect on mitochondrial function.

While demyelination is recognized as the classical target in EAE and MS, axonal and neuronal loss are now also considered as important causes of persistent clinical disability and the role of mitochondria for both processes is considered substantial [1, 2, 7, 8, 38, 46–49]. We observed that mitochondria in regions of mononuclear cellular infiltration of the cord were smaller in size. Bando et al. [50] have recently reported the disruption in the mitochondrial fusion/fission machinery in axons in EAE and shown that DLP1/Drp1, a mitochondrial fission related protein, was up-regulated while MFN1, a mitochondrial fusion-related protein, was down-regulated in EAE. Changes in mitochondrial morphology in neurons and astrocytes have also been attributed to changes in calcium dependent and independent cycling pathways [51, 52]. Mitochondrial morphology is however, dynamic and further studies that would examine changes in mitochondrial morphology at the different stages of EAE would be essential [53]. It is more important to note however, that the difference in the ratios of mitochondrial size to axonal diameter in diseased DA and resistant AO rats was not significant.

It appears in this study that demyelination at this initial stage of the disease is localized only to areas of infiltration and the present technique might not therefore, be sensitive enough to detect the effect of these limited changes on respiration in the whole of the central nervous system. Further studies using a model of chronic EAE would be required to address this concern.

The evidence from this study on the role of mitochondrial in demyelination and in EAE is therefore minimal, but it should be plausible to speculate that recovery of rats in this model, as has been observed in this and other studies [30] and in mice [1] could be due to the presence of the large population of normally functioning mitochondria in areas of the spinal cord which showed no evidence of disease.

## Conclusion

In summary, we hypothesized that the preserved cellular bioenergetics in our study might underlie the remission from disease in this model but in the light of our findings it would be more appropriate to test this hypothesis using proteolipid protein (PLP)-induced EAE in SJL/J mice. This would enable the investigation of any alterations in bioenergetics accompanying relapse and the transition to chronic EAE [54].
